# Immunomodulatory Factors Galectin-9 and Interferon-Gamma Synergize to Induce Expression of Rate-Limiting Enzymes of the *Kynurenine Pathway* in the Mouse Hippocampus

**DOI:** 10.3389/fimmu.2016.00422

**Published:** 2016-10-17

**Authors:** Alexandra K. Brooks, Marcus A. Lawson, Jennifer L. Rytych, Kevin C. Yu, Tiffany M. Janda, Andrew J. Steelman, Robert H. McCusker

**Affiliations:** ^1^Neuroscience Program, University of Illinois at Urbana-Champaign, Urbana, IL, USA; ^2^Department of Animal Sciences, University of Illinois at Urbana-Champaign, Urbana, IL, USA; ^3^Integrative Immunology and Behavior Program, University of Illinois at Urbana-Champaign, Urbana, IL, USA; ^4^Division of Nutritional Science, University of Illinois at Urbana-Champaign, Urbana, IL, USA; ^5^Department of Pathology, University of Illinois at Urbana-Champaign, Urbana, IL, USA

**Keywords:** galectin 9, IDO1, IFNγ, neuroinflammation, cytokines, kynurenine pathway

## Abstract

Elevated levels of circulating pro-inflammatory cytokines are associated with symptomology of several psychiatric disorders, notably major depressive disorder. Symptomology has been linked to inflammation/cytokine-dependent induction of the *Kynurenine Pathway*. Galectins, like pro-inflammatory cytokines, play a role in neuroinflammation and the pathogenesis of several neurological disorders but without a clearly defined mechanism of action. Their involvement in the *Kynurenine Pathway* has not been investigated. Thus, we searched for a link between galectins and the *Kynurenine Pathway* using *in vivo* and *ex vivo* models. Mice were administered LPS and pI:C to determine if galectins (Gal’s) were upregulated in the brain following *in vivo* inflammatory challenges. We then used organotypic hippocampal slice cultures (OHSCs) to determine if Gal’s, alone or with inflammatory mediators [interferon-gamma (IFNγ), tumor necrosis factor-alpha (TNFα), interleukin-1beta (IL-1β), polyinosine-polycytidylic acid (pI:C), and dexamethasone (Dex; synthetic glucocorticoid)], would increase expression of indoleamine/tryptophan-2,3-dioxygenases (DO’s: Ido1, Ido2, and Tdo2; *Kynurenine Pathway* rate-limiting enzymes). *In vivo*, hippocampal expression of cytokines (IL-1β, TNFα, and IFNγ), Gal-3, and Gal-9 along with Ido1 and Ido2 were increased by LPS and pI:C (bacterial and viral mimetics). Of the cytokines induced *in vivo*, only IFNγ increased expression of two Ido1 transcripts (Ido1-FL and Ido1-v1) by OHSCs. Although ineffective alone, Gal-9 accentuated IFNγ-induced expression of only Ido1-FL. Similarly, IFNγ induced expression of several Ido2 transcripts (Ido2-v1, Ido2-v3, Ido2-v4, Ido2-v5, and Ido2-v6). Gal-9 accentuated IFNγ-induced expression of only Ido2-v1. Surprisingly, Gal-9 alone, slightly but significantly, induced expression of Tdo2 (Tdo2-v1 and Tdo2-v2, but not Tdo2-FL). These effects were specific to Gal-9 as Gal-1 and Gal-3 did not alter DO expression. These results are the first to show that brain Gal-9 is increased during LPS- and pI:C-induced neuroinflammation. Increased expression of Gal-9 may be critical for neuroinflammation-dependent induction of DO expression, either acting alone (Tdo2-v1 and Tdo2-v2) or to enhance IFNγ activity (Ido1-FL and Ido2-v1). Although these novel actions of Gal-9 are described for hippocampus, they have the potential to operate as DO-dependent immunomodulatory processes outside the brain. With the expanding implications of *Kynurenine Pathway* activation across multiple immune and psychiatric disorders, this synergy provides a new target for therapeutic development.

## Introduction

There is a widely accepted association between activation of the immune system and major depression, with a multitude of studies showing that elevated levels of pro-inflammatory cytokines are found within the circulation of patients with MDD (1). Furthermore, up to 45% of patients receiving immunotherapy (IFNα) for hepatitis C or cancer have a greater severity of symptoms of MDD (2, 3). Extensive work using rodent models has detailed the induction of depression-like behaviors, anhedonia (decreased sucrose preference), and helplessness/despair (increased immobility during forced-swim and tail-suspension tests) after administration of LPS, pI:C, or infection with *Mycobacterium bovis* (4–7). These immune activators induce neuroinflammation; however, the mechanism(s) linking inflammation and depression is widely debated. One probable causal factor is altered tryptophan metabolism.

Elevated tryptophan metabolism to kynurenine (Kyn) *via* rate-limiting DO’s is correlated to the development of depression-like behaviors in rodent models (8) and severity of depression behaviors in patients with MDD (2). Expression of all three DO enzymes (Ido1, Ido2, and Tdo2) is increased following activation of the immune system. Their expression is increased by LPS (mimicking a bacterial infection), pI:C (mimicking a viral infection), and administration of pro-inflammatory cytokines (8–15). In contrast, in the absence of Ido1 (knockout mice), inflammation-dependent depression-like behaviors are attenuated (8, 16, 17). Inflammation-induced behavioral changes are largely attributed to increased Trp metabolism to Kyn followed by non-rate-limiting Kyn conversion to downstream neuroactive metabolites quinolinic acid (QuinA) and kynurenic acid (KynA) (5). QuinA and KynA bind to the *N*-methyl-d-aspartate and α7nAChR receptors to either enhance or decrease neurotransmitter signaling, respectively. An imbalance of these two metabolites is linked to several neurological diseases (18–20).

Galectins are β-galactoside-binding proteins. Although their mechanism of action is still unclear, Gal-1, Gal-3, and Gal-9 are believed to play significant roles in neuropathology and neuroinflammation (21). Gal-1 is typically classified as anti-inflammatory and neuroprotective: suppressing IFNγ-induced responses of microglia (22), glutamate neurotoxicity (23), and neurodegeneration (22). Gal-1 knockout mice are more susceptible to developing experimental autoimmune encephalomyelitis, a mouse model used to mimic MS symptomology. Gal-3 and Gal-9 both have context-specific immunomodulatory capabilities (24–28). As examples, Gal-3 increases secretion of pro-inflammatory cytokines (26) from microglia and astrocytes, while Gal-9 is a potent chemoattractant (29) and upregulates IFNγ production (30, 31).

A galectin–DO connection has not been directly linked to neuropsychiatric disorders, but there are several incidences of increased expression of both Gal-9 and Ido1. Increased expression of both Ido1 and Gal-9 are associated with MS (32–34), Grave’s disease, Hashimoto’s disease (35, 36), and rheumatoid arthritis (37, 38). Although the pathophysiology of these disorders is still unclear, a synergy between Ido1 and Gal-9 may be a crucial uncharacterized mechanism involved in the initiation and/or severity of immune disorders.

Data suggest that Gal-1, Gal-3, and Gal-9 modulate neuroinflammation; however, there is no evidence directly connecting galectins to the *Kynurenine Pathway*. Thus, we determined whether galectins were increased in the mouse brain following peripheral administration of LPS or pI:C. We found that both Gal-3 and Gal-9 (plus DO’s) were increased in the mouse hippocampus during neuroinflammation. We then decided to investigate whether the elevation in Gal-3 and Gal-9 were involved in the concurrent increase in expression of the rate-limiting enzymes metabolizing tryptophan to Kyn (the DO’s: Ido1, Ido2, and Tdo2) using organotypic hippocampal slice cultures (OHSCs). As we have previously shown (39), IFNγ induced Ido1 and Ido2 expression by OHSCs. Interestingly, Gal-9 was able to directly increase Tdo2 expression and increased both Ido1 and Ido2 expression in the presence of IFNγ. These are the first findings to link immunomodulatory galectin activity to the *Kynurenine Pathway*, potentially providing a new target for neuroinflammatory therapies. The ability to induce expression of the rate-limiting enzymes of the *Kynurenine Pathway* also defines a mechanism by which Gal-9 can mediate symptoms associated with several psychiatric conditions.

## Materials and Methods

### Mice

C57BL/6J mice were maintained in the University of Illinois’s Institute for Genomic Biology animal facility. Procedures and animal care were in accordance with the Guide for the Care and Use of Laboratory Animals (National Research Council) and approved by the Institutional Animal Care and Use Committee. Young adult mice, used for neuroinflammation (intraperitoneal, i.p. LPS or pI:C) experiments, were 12-week-old males at time of treatment. Saline (control), LPS, or pI:C treatments were administered prior to the start of the dark cycle. LPS (serotype 0127:B8, Sigma, St. Louis, MO, USA) was administered at 330 μg/kg body weight; this dose has been shown to elicit neuroinflammation, elevated Ido1, Ido2, and Tdo2 expression and depression-like behaviors (40). pI:C (P0913, Sigma, St. Louis, MO, USA) was administered at 12 mg/kg body weight. Previous work has shown that pI:C also causes cytokine and Ido1 response in the hippocampus and depression-like behaviors (6). Mice were sacrificed 6 h post-treatment.

### Cell Culture Reagents

Heat-inactivated horse serum (SH30074.03), Hank’s balanced salt solution (HBSS, SH30030.03), Eagle’s minimal essential medium (MEM, SH30024.02) were purchased from Hyclone (Logan, UT, USA). d-glucose (15023–021) was purchased from Gibco (Carlsbad, CA, USA). Dex (tested at 1 μM, D4902), pI:C (tested at 10 μg/ml, P0913), and Gey’s balanced salt solution (GBSS, G9779) were from Sigma (St. Louis, MO, USA). Recombinant mouse IFNγ (tested at 10 ng/ml, 315–05) was from Peprotech (Minneapolis, MN, USA). Gal-1 (1245), Gal-3 (1197), and Gal-9 (3535), all tested at 1 μg/ml and TNFα (tested at 10 ng/ml, 410-MT) were purchased from R&D Systems (Minneapolis, MN, USA). IL-1β (tested at 10 ng/ml, IL014) was purchased from Chemicon International (Temecula, CA, USA).

### Organotypic Hippocampal Slice Cultures

Organotypic hippocampal slice cultures were prepared from hippocampi of C57BL/6J pups, 7–10 days old, as previously described (39). Briefly, hippocampi were cut into 350 μm transverse slices with a McIlwain tissue chopper (Campden Instruments Ltd., UK) and positioned onto membrane inserts (PICM03050, EMD Millipore) with six slices per insert, each slice from a different animal. Inserts were placed into 6-well culture plates with 1.25 ml of medium [50% MEM, 25% horse serum, 1 × penicillin/streptomycin (Pen/Strep), 15 mM 4-(2-hydroxyethyl)-1-piperazineethanesulfonic acid (HEPES buffer, pH 7.4), 25% HBSS, and 0.5% d-glucose]. Every 2–3 days, medium was changed. On the seventh day, OHSCs were rinsed three times with serum-free medium (Dulbecco’s MEM, Pen/Strep, HEPES and 150 μg/ml bovine serum albumin) and incubated for 2 h. Slices were rinsed again with serum-free medium and treatments added to fresh serum-free medium. OHSCs were treated with IFNγ, TNFα, IL-1β, pI:C, or Dex, with or without Gal-9. To determine if effects were specific to Gal-9, independent OHSC preparations were treated with IFNγ and pI:C with or without Gal-1 and Gal-3. Six hours later, tissues and supernatants were collected and kept at −80°C for subsequent analysis.

### Reverse Transcription and Real-Time RT-PCR

RNA was extracted using the EZNA. Total RNA Kit II (Omega Bio-Tek, Norcross, GA, USA) and cDNA was prepared with the High Capacity cDNA Reverse Transcription Kit (Applied Biosystems, Grand Island, NY, USA). The cDNA was amplified to quantify steady-state gene expression by qPCR using TaqMan Universal PCR Master Mix and Prism 7900 thermocycler (Applied Biosystems, Foster City, CA, USA). Using the comparative threshold cycle method (GAPDH used to normalize target gene expression), differences in cDNA levels were determined by comparing 2^−ΔΔCts^, *C*_t_ = cycle threshold (39).

### DO Transcripts and qPCR Assay Design

**Figure 1:**
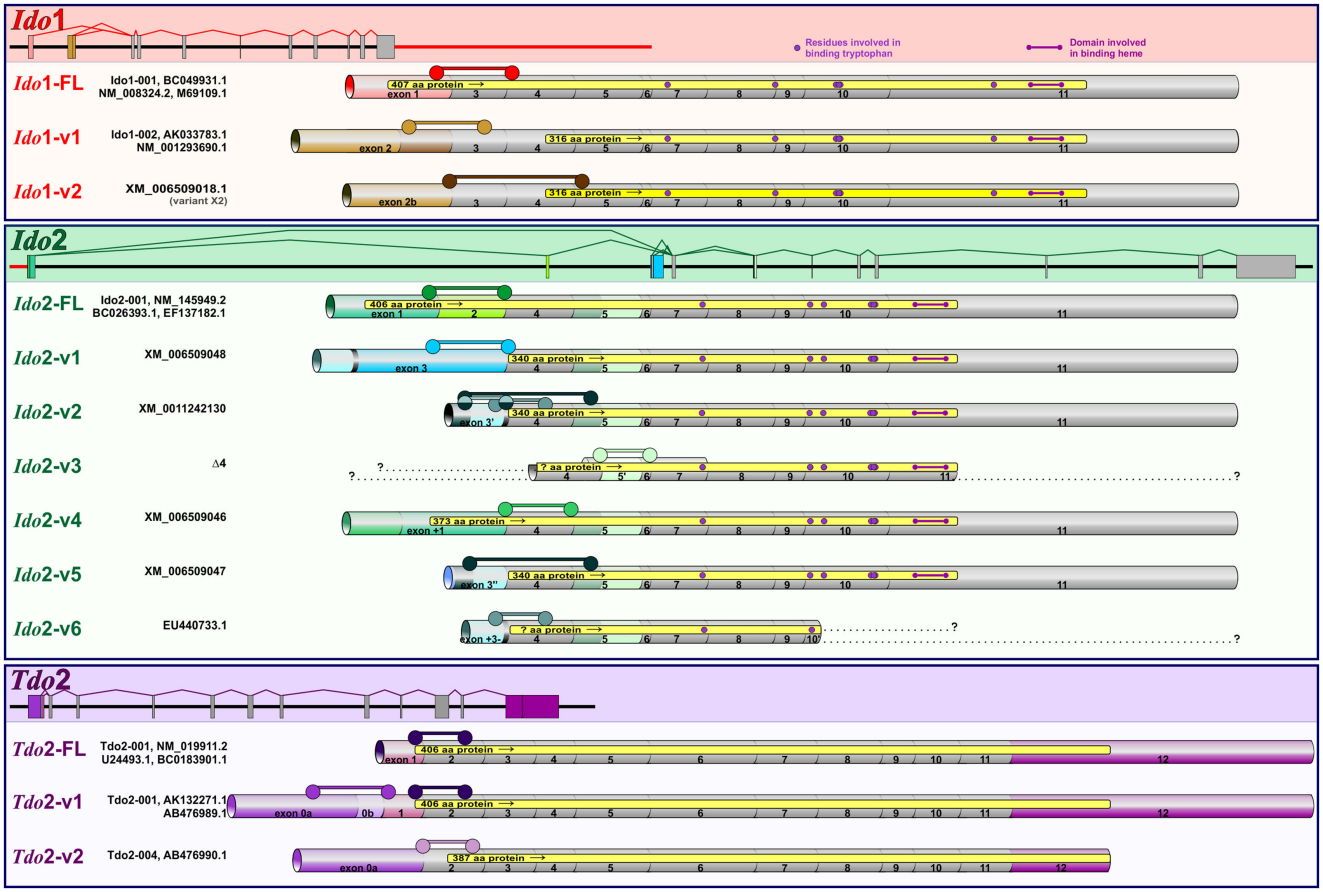
**Murine Ido1, Ido2, and Tdo2 gene structure and transcripts**. Structures of the three DO genes are shown to scale with spicing patterns illustrated for each gene. Ido1 and Ido2 are separated by 7733 bp (red line) on chromosome 8, while Tdo2 is situated on chromosome 3. As detailed previously (39), posttranslational modification of precursor mRNA results in several alternative spliced variants for each DO: full-length (FL) and variant (v) transcripts encoding variable size proteins (yellow regions within each transcript). Ensemble and NCBI databases were used to determine splicing patterns, with simplified names used in the manuscript shown to the left of database names. Within each gene, exons conserved across transcripts are shown in gray, while exons that vary by transcript are colored. Assays to quantify the expression of each transcript are described in Table 1. Exon span for each assay are shown (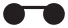).

Our previous publication describes validated qPCR assays to quantify the expression of distinct DO transcripts in mouse tissues and OHSCs. Assays for Ido1 (Ido1-FL, Ido1-v1), Ido2 (Ido2-FL, Ido2-v1, Ido2-v6, and Ido2-v3), and Tdo2 (Tdo2-FL, Tdo2-v1, and Tdo2-v2) were described (39). Gene structure for Ido1, Ido2, and Tdo2 is shown in Figure 1. Structures for the previously described transcripts are shown as well as the exon-structure of additional Ido1 (Ido1-v2) and Ido2 variants (Ido2-v2, Ido2-v4, and Ido2-v5) that were quantified for the first time for this manuscript. Assay specifics for DO transcript analyses are shown in Table 1. Probe-based assays were purchased from IDT with custom assays fashioned using the IDT PrimerQuest^®^ Design Tool. Correct amplicon size was confirmed by gel electrophoresis. Several transcripts (notably Ido1-FL) are not detectable in all naive mouse brain or control OHSC samples (*C*_t_ values “undetermined”), thus negating the ability to properly calculate relative gene expression: for mathematical analysis, a *C*_t_ value of 40.0 was assigned when this occurred.

**Table 1:** **Assay specifics for analysis of murine Ido1, Ido2, and Tdo2 steady-state gene expression**.

Transcript	Exon span	Assay ID or custom name		Primer/probe sequence	Amplicon (bp)
**Ido1-FL**	1-3-4	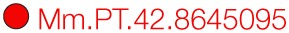	Forward	TTTGCTCTACCACATCCACTG	117
			Probe	^6-FAM^CAGATTTCT^Zen^AGCCACAAGGACCCAGG^IABkFQ^	
			Reverse	GCAGCTTTTCAACTTCTTCTCG	
**Ido1-v1**	2-3	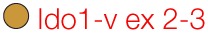	Forward	GACCCCGGACGGTAAAATTAT	138
			Probe	^6-FAM^TCGGGCAGC^Zen^TCCACATTACAATTCA^IABkFQ^	
			Reverse	TCTCAATCAGCACAGGCAG	
**Ido1-v2**	2b-3-4-5	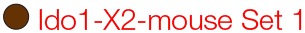	Forward	CGGACGGTGGAGCTG	256
			Probe	^6-FAM^ATTGAGAAC^Zen^GGGCAGCTTCGAGAA^IABkFQ^	
			Reverse	CGCAGTAGGGAACAGCAATA	
**Ido2-FL**	1-2-4	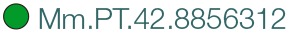	Forward	GGAGATACCACATTTCTGAGGA	114
			Probe	^6-FAM^CCAGAGGAT^Zen^TTGGAAGGAGAAAGCCAT^IABkFQ^	
			Reverse	CGATTAAGTGAGGAAGTCTGAGG	
**Ido2-v1**	3-4	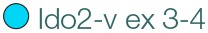	Forward	GGACTTTACATCCCTAACCTCAC	131
			Probe	^6-FAM^CCTCAGCTT^Zen^CTCGAACCCTGTAACTGTA^IABkFQ^	
			Reverse	CTGCTCACGGTAACTCTTTAGG	
**Ido2-v2**	3′-4	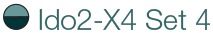	Forward	AAGCCTGCGGAGCAAAG	88
		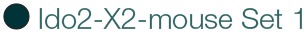	Probe	^6-FAM^TGAAGAGAT^Zen^GAGCAATGAGCCGGT^IABkFQ^	
		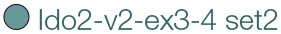	Reverse	GAGGCATCTGTCCTGCCT	
**Ido2-v3**	4-5′-6-7	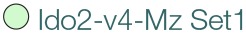	Forward	CCCCAAAAGGTATCCAGGAACT	107
			Probe	^6-FAM^CTGACCTGG^Zen^TGCTGACAAACTGGA^IABkFQ^	
			Reverse	ACTGATTTCCAACGGTCCTTCT	
**Ido2-v4**	1-4-5	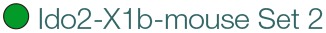	Forward	CCAAATCCTCTGATGCCTCTC	148
			Probe	^6-FAM^TAAAGAGTT^Zen^ACCGTGAGCAGCGCC^IABkFQ^	
			Reverse	AAAGGTGCTGCCAAGATCTC	
**Ido2-v5/v2**	3′-5	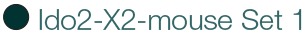	Forward	AAGCCTGCGGAGCAAAG	244/253
			Probe	^6-FAM^TGAAGAGAT^Zen^GAGCAATGAGCCGGT^IABkFQ^	
			Reverse	CCAAGTTCCTGGATACCTCAAC	
**Ido2-v6/v2**	3′-4	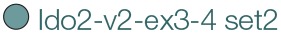	Forward	GAGCTGAAGAGATGAGCAATGA	85/85
			Probe	^6-FAM^AGGACAGAT^Zen^GCCTCTCCTGGACT^IABkFQ^	
			Reverse	ACGGTAACTCTTTAGGAATCTGC	
**Tdo2-FL/v1**	1-2	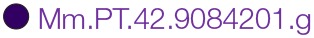	Forward	CCTGAGACACTTCAGTACTATGAG	99/99
			Probe	^6-FAM^CCCGTTTGC^Zen^AGGAAACAGTGTAGGA^IABkFQ^	
			Reverse	CTGTCTTCTTCATTGTCCTCCA	
**Tdo2-v1**	0a-0b-1	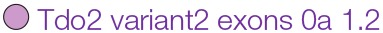	Forward	CCAGTACGAAATGAGATCCGG	132
		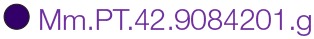	Probe	^6-FAM^AGACACAGC^Zen^CAATCAGCACCCA^IABkFQ^	
			Reverse	AGGTTTGCTAGGTCAGGAATG	
**Tdo2-v2**	0a-2	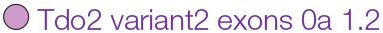	Forward	GAAATGAGATCCGGGCTAAGAG	78
			Probe	^6-FAM^TGGGTGCTG^Zen^ATTGGCTGTGTCT^IABkFQ^	
			Reverse	GTGTATCTTTTATGTATCCTGATTGCC	

*Probe-based qPCR assays were purchased from IDT. The IDT PrimerQuest^®^ Design Tool was used to design custom assays. Assay specifics: location of exons spanned, assay ID, primer/probe sequences, and amplicon size (confirmed by gel electrophoresis) are shown. The transcript Ido2-v2 is detected by three assays: Ido2-X4 Set 4, Ido2-X2-mouse Set 1, and Ido2-v2-ex3-4 set2. However, the assay Ido2-X4 Set 4 spans an exon boundary unique to the Ido2-v2 transcript. Discerning changes in Ido2-v2 expression is possible using this assay. In addition, since the assays that assess Ido2-v5/v2 and Ido2-v6/v2 also detect Ido2-v2, changes in Ido2-v5 and Ido2-v6 can only be estimated based on unique changes in gene expression using either Ido2-X2-mouse Set 1 or Ido2-v2-ex3-4 set2 compared to Ido2-X4 Set 4. Similarly, the entire Tdo2-FL sequence in found within Tdo2-v1, and as such, it is not possible to generate an assay specific to Tdo2-FL. However, the current data and our published work (39) strongly indicate that these transcripts are differentially regulated*.

*Colored fonts are used to match the gene coloration in the graphic Figure 1 and data figures throughout the manuscript*.

### Statistics

Data are reported as mean ± SEM for three to four independent OHSC preparations per treatment combination and six mice/treatment group for the adult animal study. SigmaPlot 13.0 software was used to conduct two-way analysis of variance (two-way ANOVA) using a 2 × 6 (OHSC) or 2 × 2 (*in vivo*) arrangement of treatments. In the presence of a significant interaction, assessed by Holm–Šídák method, *post hoc* analyses for multiple comparisons were performed. Comparisons were considered significant at *p* < 0.05.

## Results

### *In Vivo*: Neuroinflammation

#### LPS and pI:C Induce DOs, Cytokines, and Galectins in the Mouse Hippocampus

##### Ido1 Expression

**Figure 2:**
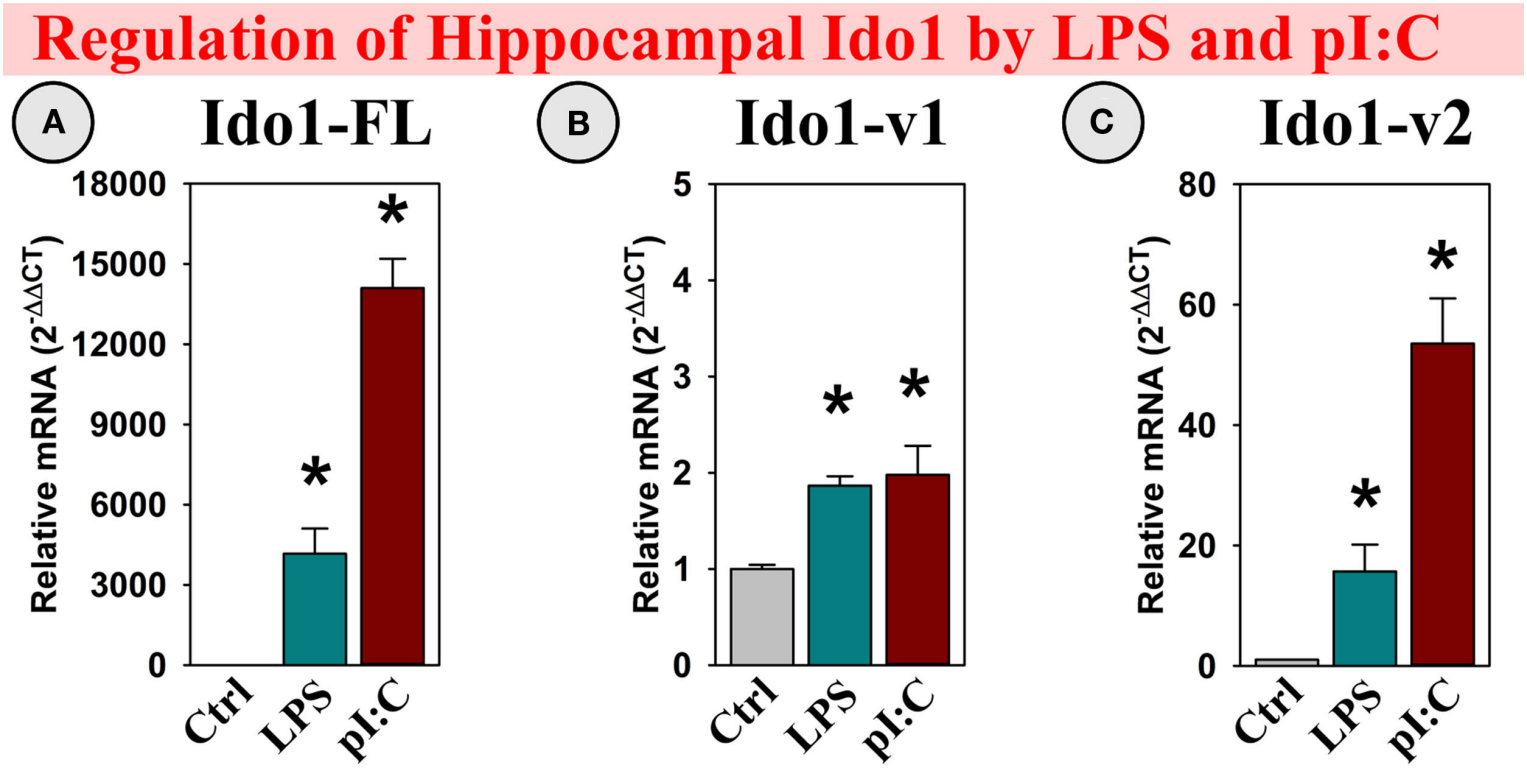
***In vivo*, hippocampal Ido1 expression is increased after LPS and pI:C administration**. Mice were administered saline (Ctrl), LPS, or pI:C and hippocampi collected after 6 h. Gene expression of three Ido1 transcripts was measured. Expression levels are relative to Ctrl samples normalized to 1.0. *p < 0.05 for the effect of LPS or pI:C. Average Ctrl *C*_t_ values for each transcript: **(A)** Ido1-FL: *C*_t_ = 40.0, **(B)** Ido1-v1: *C*_t_ = 29.8, and **(C)** Ido1-v2: *C*_t_ = 39.7. *C*_t_ values indicate that Ido1-FL and Ido1-v2 are essentially not expressed in naive (Ctrl) mouse hippocampi, whereas Ido1-v1 expression is detectable in all samples.

LPS and pI:C were injected intraperitoneally into adult mice to determine if, along with the DO’s, brain galectin expression was regulated by neuroinflammation. Similar to previous studies (6, 13, 40), hippocampal Ido1 expression was increased within several hours of a LPS or pI:C challenge. Both LPS and pI:C induced all three Ido1 transcripts: Ido1-FL, Ido1-v1, and Ido1-v2 (all *p* < 0.01; Figures 2A–C), with the greatest fold increase seen for Ido1-FL followed by Ido1-v2 (both transcripts essentially absent in the hippocampus of naive/control mice), but only a minor increase for the highly expressed Ido1-v1 (see relative basal *C*_t_ values in the figure legend).

##### Ido2 Expression

**Figure 3:**
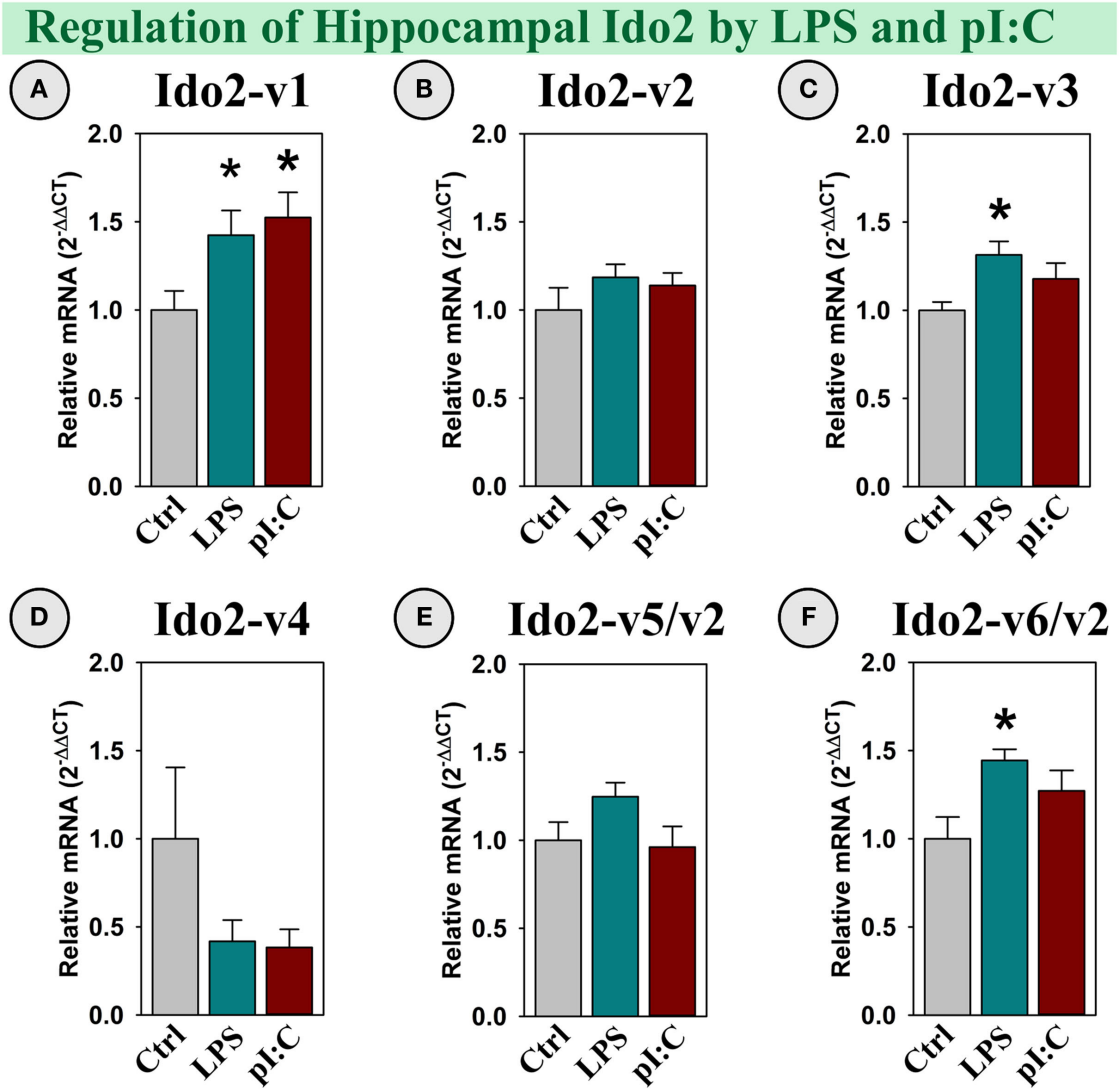
**Specific Ido2 transcripts expressed in the mouse hippocampus are increased by LPS and pI:C *in vivo***. Mice were administered saline (Ctrl), LPS, or pI:C and hippocampi were collected after 6 h. Expression of six Ido2 gene transcripts was measured. Expression levels are relative to Ctrl samples set to 1.0. *p < 0.05 for the effect of LPS or pI:C. Average Ctrl *C*_t_ values for each transcript: **(A)** Ido2-v1: *C*_t_ = 29.2, **(B)** Ido2-v2: *C*_t_ = 28.9, **(C)** Ido2-v3: *C*_t_ = 28.4, **(D)** Ido2-v4: *C*_t_ = 38.5, **(E)** Ido2-v5/-v2: *C*_t_ = 31.6, and **(F)** Ido2-v6/-v2: *C*_t_ = 28.3. *C*_t_ values indicate that Ido2-FL (non-detectable, not shown) and Ido2-v4 are essentially not expressed in naive (Ctrl) mouse hippocampi. Expression of other Ido2 transcripts are readily detectable in all samples.

Several Ido2 transcripts are expressed in the mouse brain. Ido2-v1, Ido2-v3, and Ido2-v6 were increased in the hippocampus following LPS treatment (all *p* < 0.05; Figures 3A,C,F), whereas only Ido2-v1 was elevated by pI:C (*p* < 0.05; Figure 3A). Using assays that specifically assess Ido2-FL, Ido2-v2, Ido2-v4, and Ido2-v5 (Table 1), we found that their expression was not induced by either LPS or pI:C (Figures 3B,D,E). Ido2-FL was not detectable nor induced (not shown).

##### Tdo2 Expression

**Figure 4:**
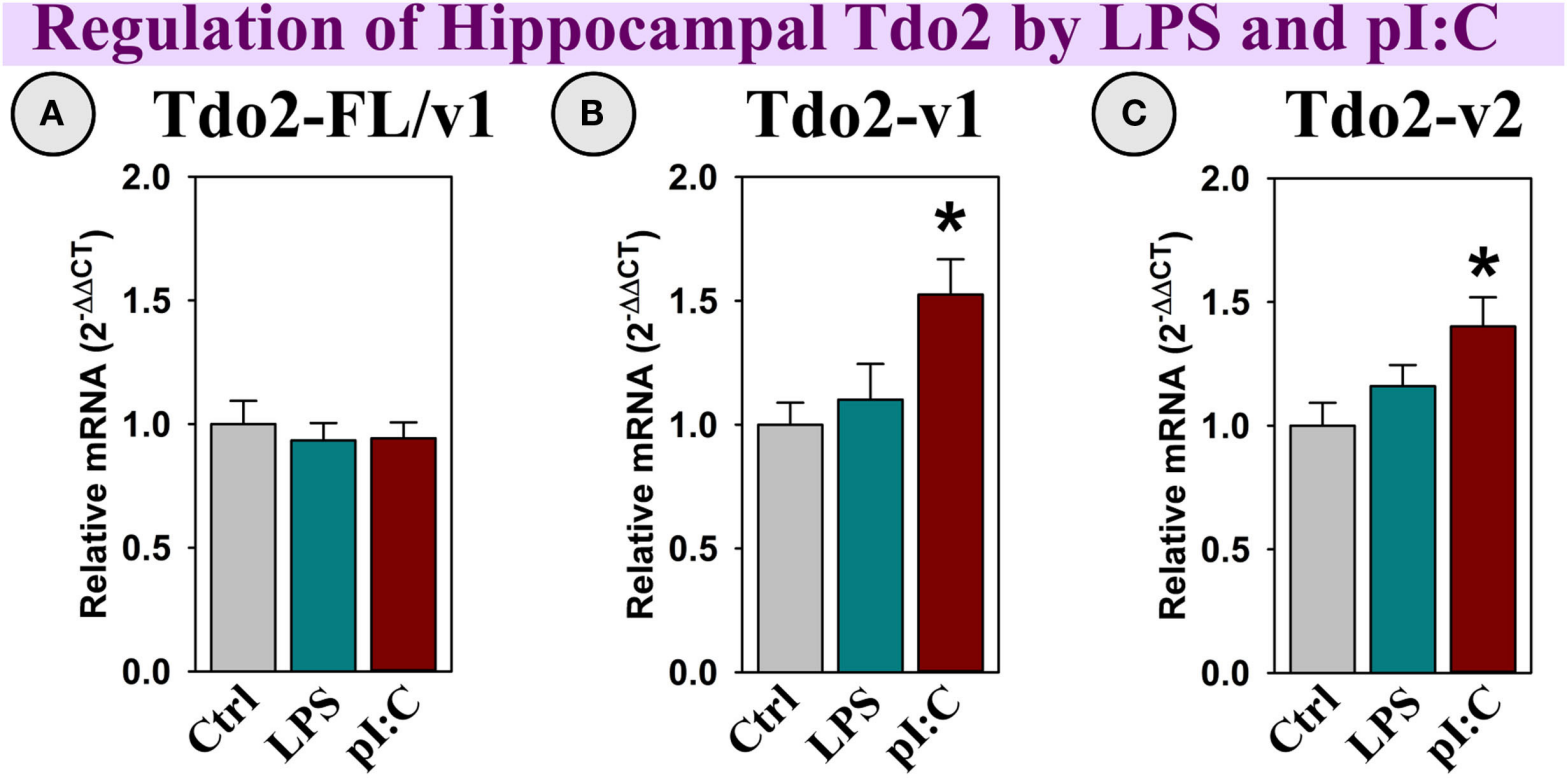
***In vivo*, hippocampal Tdo2 expression is increased after pI:C administration**. Mice were administered saline (Ctrl), LPS, or pI:C and hippocampi were collected after 6 h. Gene expression of three Tdo2 transcripts was measured. Expression is relative to Ctrl, normalized to 1.0. *p < 0.05 for the effect of pI:C. Average Ctrl *C*_t_ values for each transcript: **(A)** Tdo2-FL: *C*_t_ = 21.4, **(B)** Tdo2-v1: *C*_t_ = 28.7, and **(C)** Tdo2-v2: *C*_t_ = 27.7. *C*_t_ values indicate that all three Tdo2 transcripts are well-expressed in the mouse hippocampus.

Tdo2-FL expression was not altered by LPS or pI:C (Figure 4A); however, both Tdo2-v1 and Tdo2-v2 were increased by pI:C (*p* < 0.05, Figures 4B,C).

##### Cytokines

**Figure 5:**
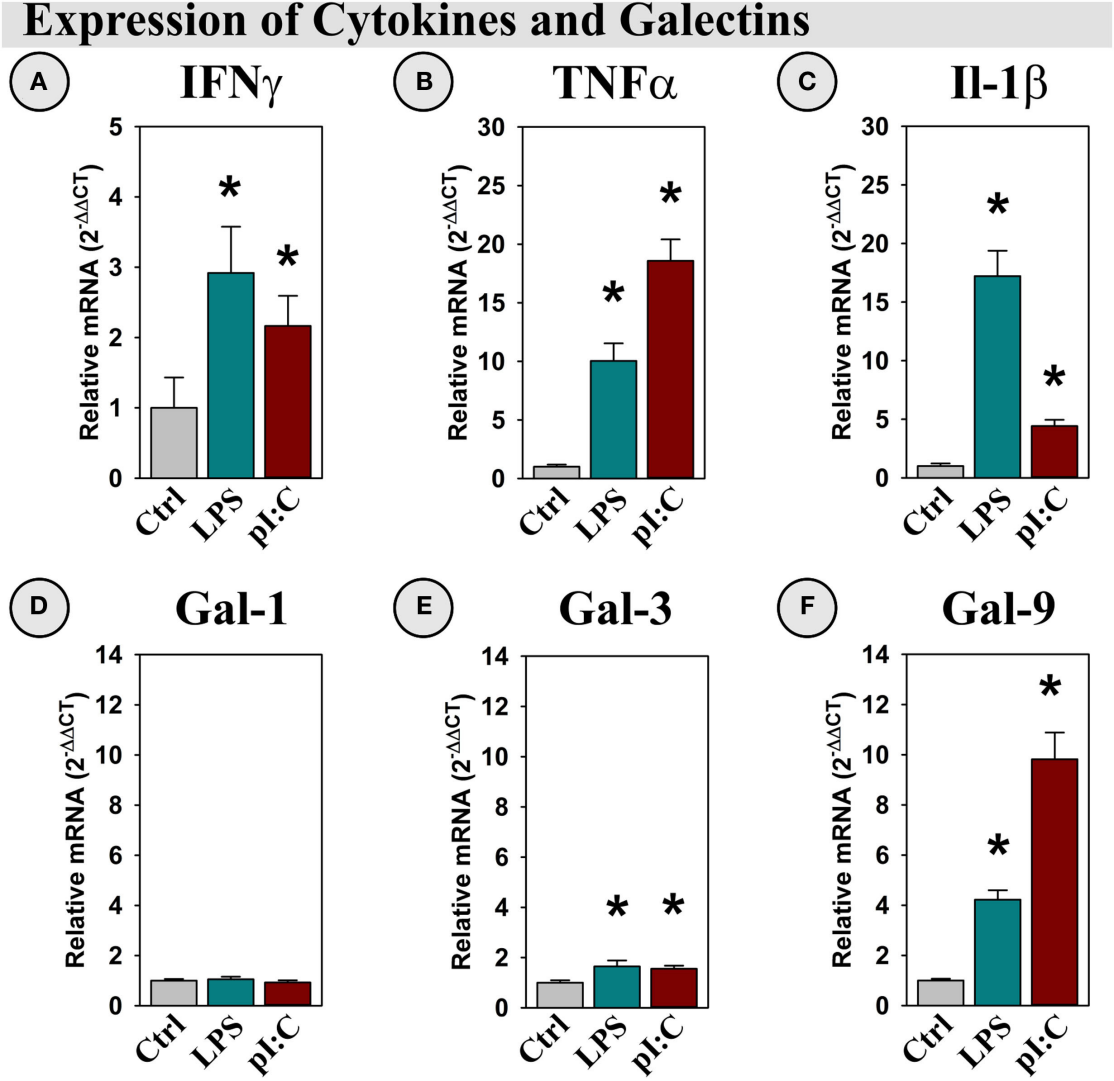
**LPS and pI:C increase expression of pro-inflammatory cytokines and galectins in the mouse hippocampus**. Mice were administered saline (Ctrl), LPS, or pI:C and hippocampi were collected after 6 h. Gene expression of **(A)** IFNγ, **(B)** TNFα, **(C)** IL-1β, **(D)** Gal-1, **(E)** Gal-3, and **(F)** Gal-9 was measured and expression is shown relative to Ctrl. *p < 0.05 for main effect of LPS or pI:C.

Illustrating a classical neuroinflammatory response (13, 41), both LPS and pI:C increased IFNγ, TNFα, and IL-1β expression in the mouse hippocampus (all *p* < 0.05; Figures 5A–C). Gal-1 was expressed, but not induced by LPS or pI:C (Figure 5D), while Gal-3 expression was doubled by LPS and pI:C (*p* < 0.05, Figure 5E). Interestingly, Gal-9 expression was increased ~ 4-fold by LPS and ~10-fold by pI:C (all *p* < 0.001, Figure 5F).

##### *In Vivo* Summary

These data illustrate that neuroinflammation increased the expression of Gal-3 and Gal-9 along with the expected increase in pro-inflammatory cytokines. Galectin and cytokine induction was accompanied by elevated DO expression within the mouse hippocampus, but in a DO and transcript-specific manner. Thus, we next investigated which (if any) of the induced cytokines or galectins directly mediated the changes in hippocampal DO expression, using OHSCs.

### *Ex Vivo*: OHSCs

#### Gal-9 Synergized with IFNγ to Induce Ido1-FL Expression

**Figure 6:**
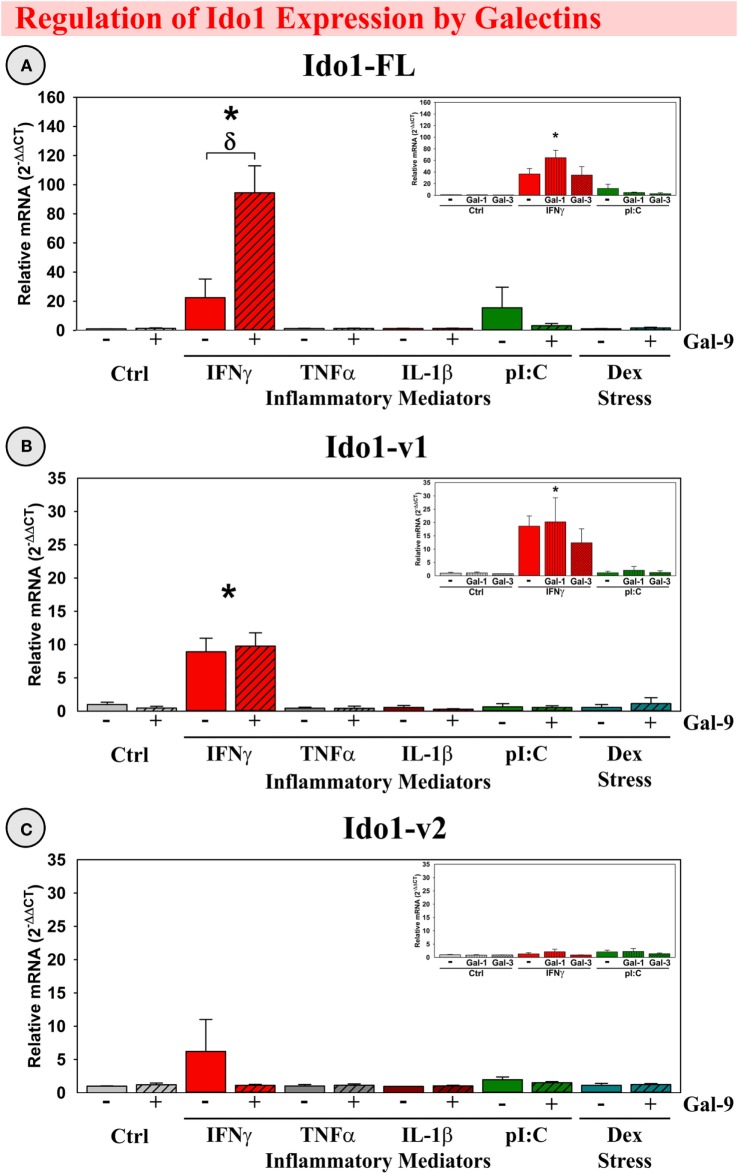
**IFNγ increases expression of two Ido1 transcripts with Gal-9 interacting to accentuate only Ido1-FL *ex vivo***. OHSCs were treated with IFNγ, TNFα, IL-1β, pI:C, and Dex ± Gal-9. Additional OHSCs (insets) were treated with IFNγ and pI:C ± Gal-1 or Gal-3. Gene expression of three Ido1 transcripts was measured and expression levels are relative to Ctrl samples normalized to 1.0. *p < 0.05 for the effect of IFNγ.^δ^ *p* < 0.05 for Gal-9 within inflammatory mediator. Average Ctrl *C*_t_ values for each transcript: **(A)** Ido1-FL: *C*_t_ = 40.0, **(B)** Ido1-v1: *C*_t_ = 34.9, and **(C)** Ido1-v2: *C*_t_ = 40.0. *C*_t_ values indicate that only Ido1-v1 is expressed in Ctrl slice cultures, paralleling the relative expression pattern of mouse hippocampi (Figure 2).

As shown previously, IFNγ induced Ido1-FL (9, 39) and Ido1-v1 expression (39) by OHSCs (*p* < 0.001; Figures 6A,B). Gal-9 alone did not induce expression of any Ido1 transcript; however, Gal-9 significantly interacted with IFNγ to accentuate IFNγ-induced Ido1-FL expression [*F*_(5,24)_ = 8.02, MSE = 1418, *p* < 0.001, Figure 6A]. In contrast, Ido1-v2 was not induced by IFNγ or Gal-9 (Figure 6C), despite being inducible *in vivo*.

Based on our *in vivo* results showing Gal-1 and Gal-3 expression within mouse hippocampus, we tested for Gal-9 specificity by treating OHSCs with Gal-1 or Gal-3. Surprisingly, considering their immunomodulatory capabilities, Gal-1 and Gal-3 did not alter Ido1 expression alone or in the presence of IFNγ (Figure 6, insets), suggesting that the synergy with IFNγ was Gal-9-specific. An additional degree of specificity is illustrated by the inability of TNFα, IL-1β, pI:C, or Dex to induce any Ido1 transcript or to synergize with galectins. Thus, the elevated expression of IFNγ and Gal-9 associated with neuroinflammation *in vivo* would be expected to act synergistically to enhance Ido1-FL expression.

#### Gal-9 Synergized with IFNγ to Induce the Expression of Ido2-v1

**Figure 7:**
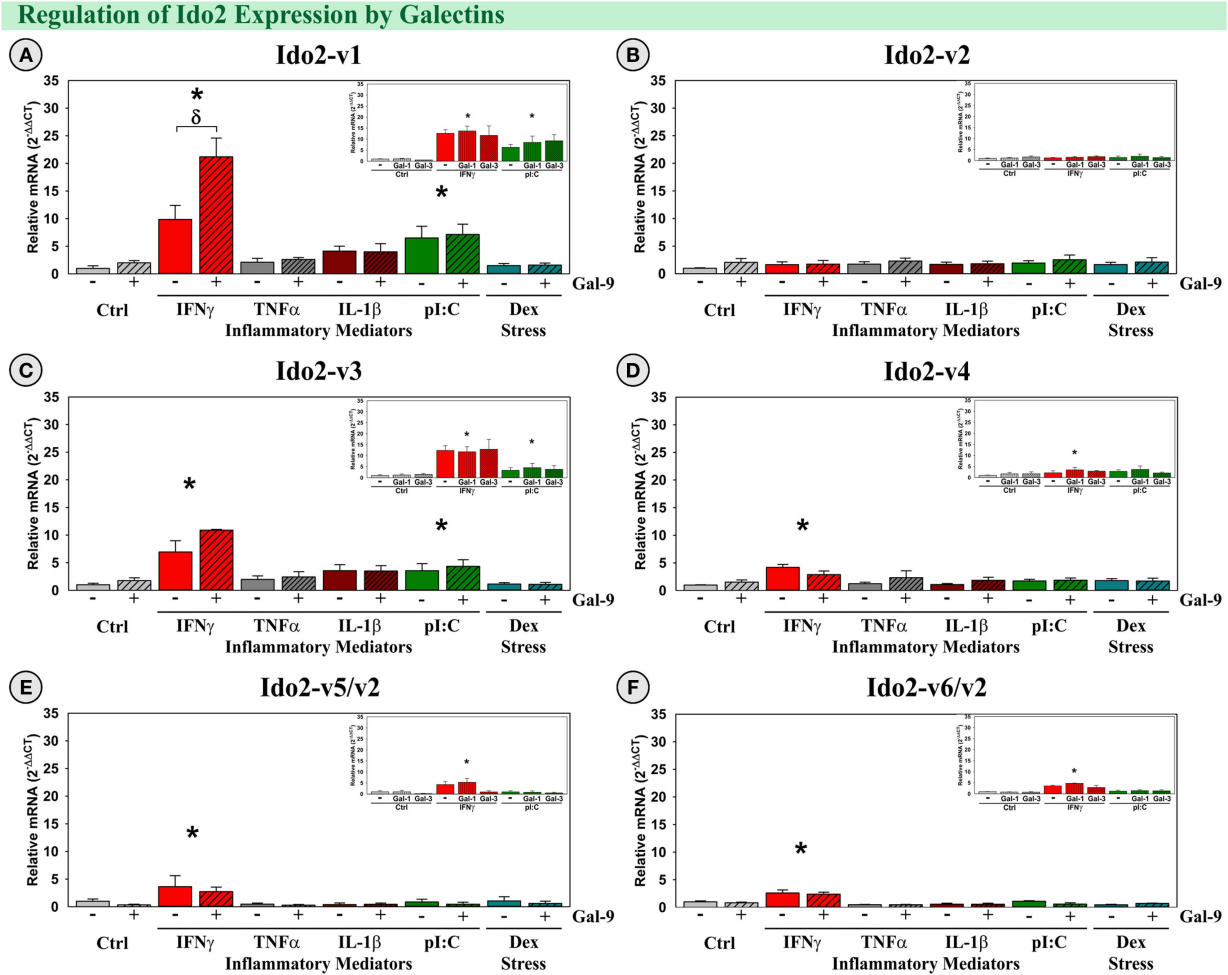
**IFNγ increases expression of five Ido2 transcripts with Gal-9 interacting to accentuate only Ido2-v1 *ex vivo***. OHSCs were treated with IFNγ, TNFα, IL-1β, pI:C, and Dex ± Gal-9. Additional OHSCs (insets) were treated with IFNγ and pI:C ± Gal-1 or Gal-3. Gene expression of six Ido2 transcripts was measured, and expression levels are relative to Ctrl’s normalized to 1.0. *p < 0.05 for the effect of IFNγ.^δ^ *p* < 0.05 effect of Gal-9 within inflammatory mediator. Average Ctrl *C*_t_ values for each transcript: **(A)** Ido2-v1: *C*_t_ = 32.9, **(B)** Ido2-v2: *C*_t_ = 30.6, **(C)** Ido2-v3: *C*_t_ = 34.4, **(D)** Ido2-v4: *C*_t_ = 40.0, **(E)** Ido2-v5/-v2: *C*_t_ = 36.1, and **(F)** Ido2-v6/-v2: *C*_t_ = 33.7. *C*_t_ values indicate that Ido2-FL (non-detectable, not shown) and Ido2-v4 are not expressed in Ctrl slice cultures, similar to the hippocampus of naive (Ctrl) mice (Figure 3).

Similar to our previous publication (39), both IFNγ and pI:C induced expression of Ido2-v1 and Ido2-v3 (*p* < 0.001; Figures 7A,C), but only IFNγ increased expression of Ido2-v6/v2 (*p* < 0.001; Figure 7F). Interestingly, Gal-9 synergized with IFNγ to accentuate only Ido2-v1 expression [*F*_(5,24)_ = 4.02, MSE = 30.0, *p* < 0.01, Figure 7A]. Three additional Ido2 variants were expressed by OHSCs. IFNγ induced Ido2-v4 and Ido2-v5 expression (*p* < 0.05, Figures 7D,E), but not Ido2-v2 (Figure 7B). These data suggest that each Ido2 transcript is differentially regulated. In addition, neither TNFα, IL-1β, nor Dex increased Ido2 expression, alone or with Gal-9. Gal-1 and Gal-3 did not affect Ido2 expression (Figure 7; insets). Thus, the elevated expression of IFNγ and Gal-9 associated with neuroinflammation *in vivo* would be expected to act synergistically to enhance Ido2-v1 expression.

#### Gal-9 Induced Tdo2-v1 and Tdo2-v2

**Figure 8:**
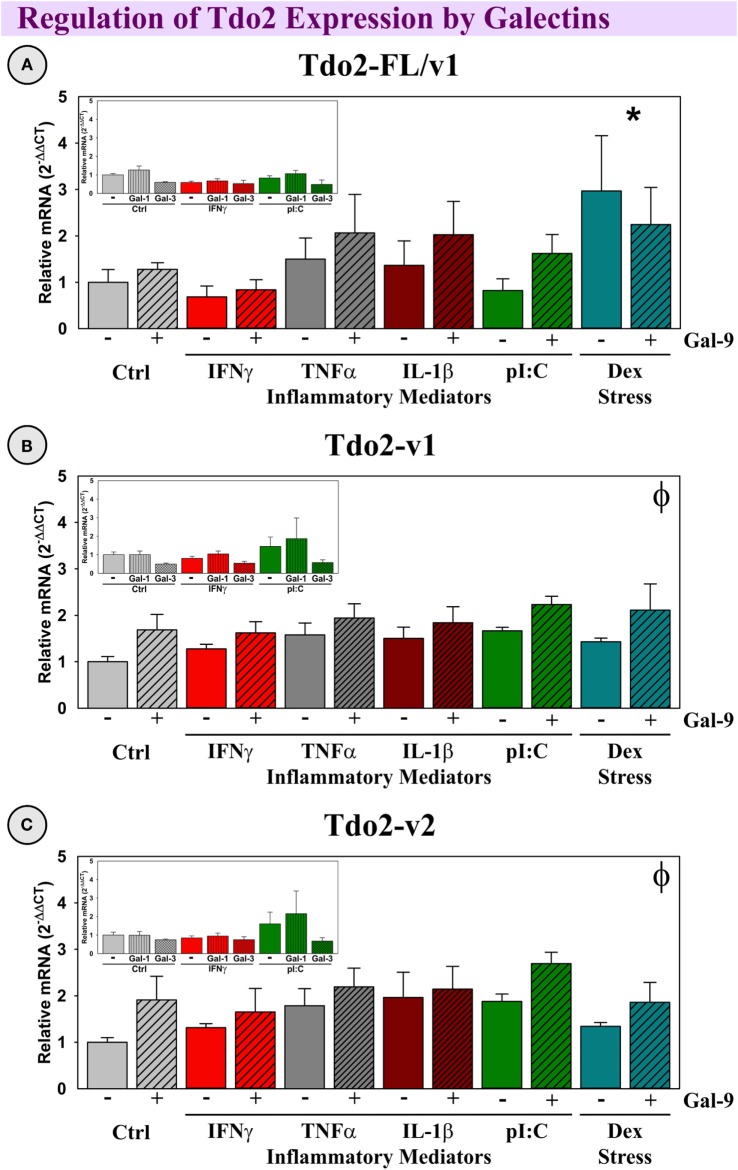
**Dex increases Tdo2-FL, whereas Gal-9 increases Tdo2-v1 and Tdo2-v2 expression *ex vivo***. OHSCs were treated with IFNγ, TNFα, IL-1β, pI:C, and Dex ± Gal-9. Additional OHSCs (insets) were treated with IFNγ and pI:C ± Gal-1 or Gal-3. Gene expression of Tdo2 transcripts was measured and levels are normalized to 1.0 for Ctrl within each transcript. *p* < 0.05 for the effect of Dex. ^ϕ^*p* < 0.05 for the main effect of Gal-9. Average Ctrl *C*_t_ values for each transcript: **(A)** Tdo2-FL: *C*_t_ = 29.1, **(B)** Tdo2-v1: *C*_t_ = 26.3, and **(C)** Tdo2-v2: *C*_t_ = 25.3. *C*_t_ values indicate that all three Tdo2 transcripts are well-expressed in mouse hippocampal slice cultures, paralleling their expression pattern in the naive mouse hippocampus (Figure 4).

All three Tdo2 transcripts are well-expressed by OHSCs (Figure 8, *C*_t_’s presented in figure legend). Neither IFNγ, TNFα, IL-1β nor pI:C affect Tdo2 expression (all three transcripts), although Dex has a specific inductive action on the Tdo2-FL transcript [*p* < 0.05, Figure 8A; as previously reported (39)]. Gal-9 did not affect the expression of Tdo2-FL, but Gal-9 slightly, and significantly increased expression of both Tdo2-v1 and Tdo2-v2 (*p* < 0.05; Figures 8B,C). Neither Gal-1 nor Gal-3, alone or in combination with inflammatory mediators, affected Tdo2 expression (Figure 8, insets). Thus, the elevated expression of Gal-9 associated with neuroinflammation *in vivo* would be expected to enhance Tdo2-v1 and Tdo2-v2 expression within the hippocampus; whereas Tdo2-FL is specifically induced by the glucocorticoid receptor agonist, Dex.

#### Gal-9 Increased TNFα and IL-6 Expression

**Figure 9:**
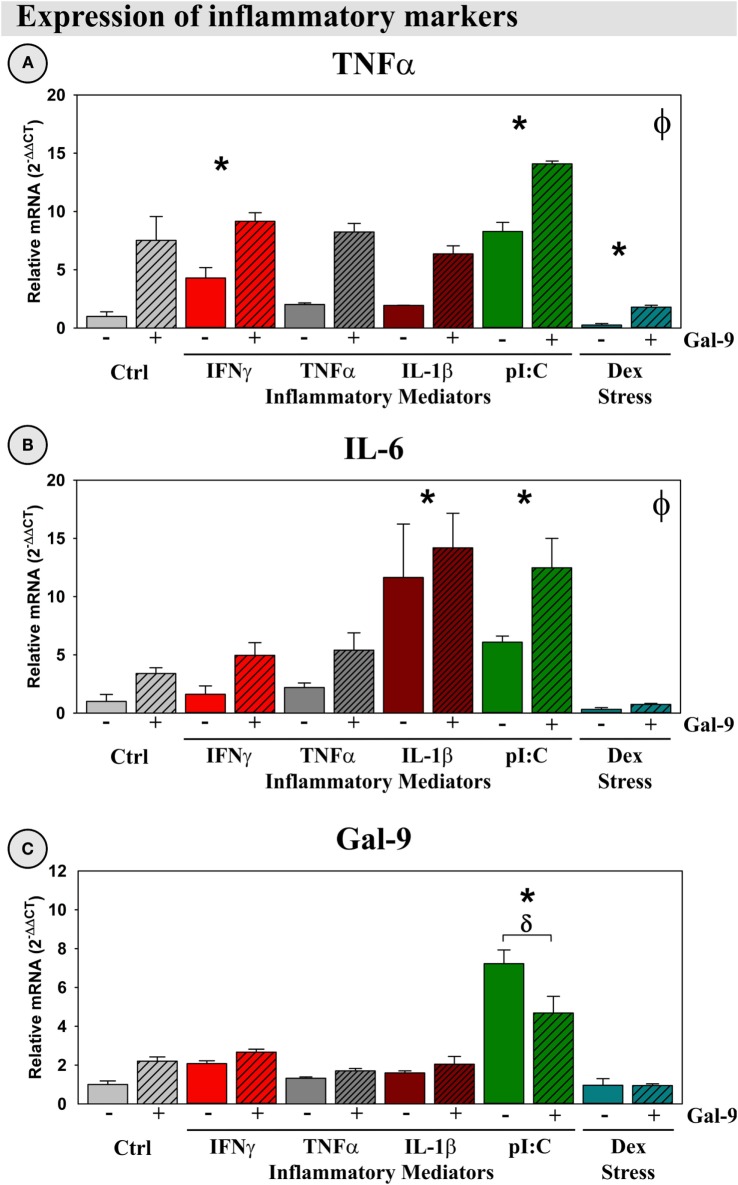
**Gal-9 increases expression of pro-inflammatory mediators and downregulates its own expression *ex vivo***. OHSCs were treated with IFNγ, TNFα, IL-1β, pI:C, and Dex ± Gal-9. Expression of **(A)** TNFα, **(B)** IL-6, and **(C)** Gal-9 was quantified. Expression for Ctrl cultures was normalized to 1.0. *p < 0.05 for the effects of IFNγ, pI:C, IL-1β, and Dex. ^ϕ^*p* < 0.05 for the main effect of Gal-9. ^δ^*p* < 0.05 effect for Gal-9 within inflammatory mediator. IFNγ expression was not detected in OHSCs.

Previous work had shown that Gal-9 can increase production of pro-inflammatory cytokines, such as TNFα from microglia (24). To validate this immunomodulatory action, we investigated the ability of our treatments, including Gal-9, to induce an inflammatory response by OHSCs. Gal-9, IFNγ, and pI:C induced TNFα expression (*p* < 0.05; Figure 9A), while Gal-9, pI:C, and IL-1β induced IL-6 expression (*p* < 0.05; Figure 9B). As expected, Dex decreased TNFα expression, acting in a typical anti-inflammatory (glucocorticoid-like) manner (*p* < 0.05). Only pI:C increased expression of Gal-9 (*p* < 0.05); this induction was diminished by Gal-9 (*p* < 0.05; Figure 9C). Of note, none of the treatments (or combinations) resulted in detectable expression of IFNγ by OHSCs (not shown). Thus, IFNγ responsible for Ido1 and Ido2 expression is not derived from cells resident in the hippocampal parenchyma, while, in contrast, Gal-9 is expressed by the brain.

## Discussion

We previously reported an inflammation-by-stress synergy (IFNγ × glucocorticoid) that functions to accentuate expression of Ido1-FL, Ido1-v1, and Ido2-v3 within the mouse hippocampus (39). We now report another exciting synergistic interaction between two immunomodulatory factors: IFNγ and Gal-9 that acts to enhance Ido1-FL and Ido2-v1 expression. These data indicate that IFNγ drives Ido1 and Ido2 induction, but secondary factors determine which transcripts mediate the synergistic induction of the Ido’s. We also describe assays to quantify expression of multiple Ido1 and Ido2 transcripts *in vivo* (during neuroinflammation) and *ex vivo* (during inflammatory challenges of OHSCs).

*In vivo*, LPS or pI:C induced neuroinflammation (i.e., increased cytokine expression) and galectin expression, which was accompanied by increased expression of all Ido1 transcripts, but only specific Ido2 and Tdo2 transcripts. This is the first report demonstrating that both LPS- and pI:C-induced neuroinflammation involved increased Gal-3 and Gal-9 expression: paralleling the elevated expression of IFNγ, TNFα, and IL-1β. Thus, we treated OHSCs with these cytokines ± galectins to determine which factors (or combination thereof) were responsible for elevated DO expression *in vivo*. Using OHSCs, we found that Gal-9 acted independently to increase Tdo2-v1 and Tdo2-v2 expression, but Gal-9 only increased expression of Ido1 and Ido2 in the presence of IFNγ. These findings suggest that Gal-9 plays a previously undefined role in the induction of the *Kynurenine Pathway*.

### Ido1

We previously detailed the regulation of two Ido1 transcripts and compared their relative expression across multiple areas of the brain. Ido1-v1 was well expressed (39) in the mouse hippocampus and other brain regions, whereas Ido1-FL is low/undetectable across the naive mouse brain (39). Like Ido1-FL, we now show that a third Ido1 transcript, Ido1-v2, is low/undetectable in the mouse hippocampus of naive mice (Figure 2). Not only is hippocampal basal expression of these three Ido1 transcripts different but also the regulation of Ido1 transcripts by inflammatory mediators and corticosteroids is unique as well. Expression of both Ido1-FL and Ido1-v2 were induced by LPS and pI:C *in vivo* (current work), but not *ex vivo* (39), suggesting that *in vivo* hippocampal responses to LPS and pI:C must be mediated by other secondary factors: presumably, cytokines and other immunomodulatory mediators. Using OHSCs, we found that only IFNγ was able to directly induce Ido1-FL and Ido1-v1 expression [Figure 6; (39)]. Since brain/hippocampal IFNγ is inducible by LPS and pI:C, but OHSCs did not express IFNγ (not shown), non-resident cells from the periphery are probably responsible for the elevated IFNγ expression within the brain that occurs *in vivo* and thus for the increase in Ido1 expression. Indeed, during neuroinflammation, IFNγ^+^ cells infiltrate the brain (42). Hippocampal Ido1-v2 expression is increased by LPS and pI:C *in vivo*; however, we have not, as yet, identified the mechanism responsible for this response; none of the treatments including IFNγ increased Ido1-v2 expression by OHSCs.

**Figure 10:**
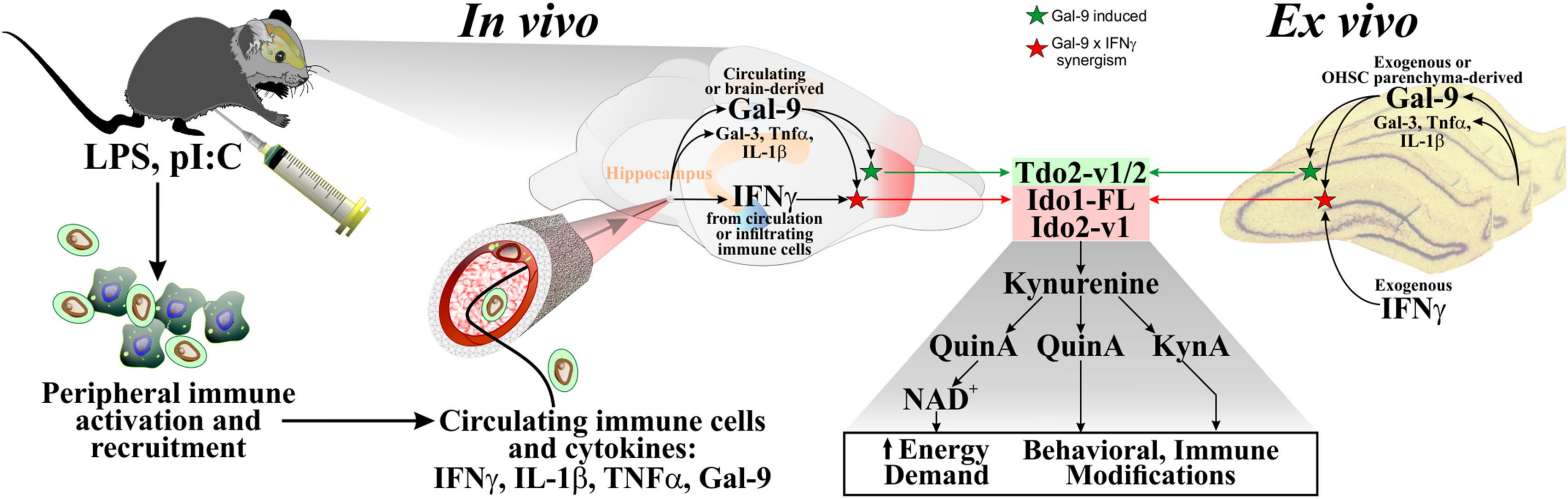
**Proposed model for *Kynurenine Pathway* activation in the brain**. Multiple studies have shown that *in vivo* administration of LPS or pI:C to mice increased peripheral pro-inflammatory cytokines (including IFNγ, TNFα, and IL-1β). These circulating cytokines, as well as immune cells that produce them, can enter the brain, to stimulate brain parenchymal production of additional immunomodulatory agents (cytokines and galectins). Gal-9 (produced within the brain or from the circulation) and IFNγ (from the circulation or infiltrating immune cells) synergize to increase expression of DO transcripts in the brain. *Ex vivo*, IFNγ is added [since brain parenchyma (i.e., OHSCs) does not express this cytokine], Gal-9 is also added to directly test its activity (but Gal-9 is expressed by cells resident to the brain). Independent of the source, IFNγ and Gal-9 increase DO expression in a DO- and transcript-specific manner, modeling *in vivo* responses. DO induction would increase flux down the *Kynurenine Pathway* to serve two purposes: supplying NAD^+^ for the brain, but with consequences (metabolites, Kyn, KynA, and QuinA, modulate behavior and immune activity).

In the naive hippocampus and control OHSCs, only Ido1-v1 is readily detectable, but Ido1-v1 expression is far less sensitive to pro-inflammatory induction compared to Ido1-FL. These data suggest that Ido1-v1 probably has a major role in basal metabolism [supplying Kyn for nicotinamide/NAD^+^ synthesis (43)], whereas the sizeable induction of Ido1-FL by IFNγ plus either Gal-9 (current work) or glucocorticoids (39) is necessary to meet the increased energy/NAD^+^ demand associated with inflammation (44–46) or stress, respectively (Figure 10). Indeed, elevated central nervous system NAD^+^ levels during experimental MS (47) are associated with increased Ido1 (48). An unfortunate consequence of Ido1 induction is elevated production and release of downstream neuroactive Kyn metabolites (QuinA and KynA) that mediate depression-like, anxiogenic, and adverse cognitive behaviors (49).

### Ido2

We recently found that several Ido2 transcripts are differentially expressed across the mouse brain, and again, inflammatory mediators and corticosteroids differentially regulate Ido2 transcripts (39). Obviously, posttranslational processing of Ido2 (and Ido1) is not a random process but is utilized to fine-tune mRNA expression. A switch in posttranslational processing of Ido2 was first addressed using macrophages and B-cells, wherein both cell types can switch from expression of predominantly Ido2-FL to predominantly Ido2-v3 (50). While investigating Ido2 expression in the brain, we had first shown that LPS increased hippocampal Ido2 expression *in vivo*, using an assay that simultaneously quantified all Ido2 transcripts (40). In the current work, we refined our understanding of central Ido2 expression by quantifying the regulation of its various transcripts. During *in vivo* neuroinflammation, LPS and pI:C increased hippocampal Ido2 expression in a transcript-specific manner. Both LPS and pI:C increased Ido2-v1 expression; however, only LPS increased Ido2-v3 and Ido2-v6/v2. Since LPS and pI:C were administered intraperitoneally, we used OHSCs to identify direct versus indirect mechanisms responsible for Ido2 induction.

Together with our previous work (39), OHSC expression of Ido2-v1 and Ido2-v3 was increased by IFNγ, LPS, and pI:C, while Ido2-v4, Ido2-v5, and Ido2-v6 were increased slightly but by only IFNγ. These data indicate that LPS and pI:C may act directly or *via* IFNγ to induce Ido2-v1 and Ido2-v3 within the hippocampus. In contrast, *in vivo* induction of Ido2-v4, Ido2-v5, and Ido2-v6 are mediated by IFNγ. Uniquely, Ido2-v2 is highly expressed, but unaffected by inflammatory mediators and galectins. Overall, in both the mouse hippocampus and OHSCs, Ido2-v1 and Ido2-v3 are most susceptible to induction by inflammation, whereas the other transcripts are either unchanged *in vivo* or modestly induced *ex vivo*. These data suggest that Ido2-v1 and Ido2-v3 induction was necessary to increase flux down the *Kynurenine Pathway* (Figure 10) to meet the increased energy (NAD^+^) demand associated with inflammation (44–46). A repercussion of their induction may be altered behaviors associated with Kyn production (50) and conversion to QuinA or KynA (20). Other Ido2 transcripts are relatively intractable and involved in basal metabolism.

### Gal-9

Excitingly, in the presence of IFNγ, Gal-9 accentuated Ido1-FL and Ido2-v1 expression by OHSCs; neither Gal-1 nor Gal-3 affected DO expression. This statistical interaction suggests that Ido1 and Ido2 are regulated in a transcript-specific manner by additional factors: glucocorticoids (39) and Gal-9 (current work). Although IFNγ initiates Ido1-FL and Ido2-v1 induction [and indeed IFNγ action is necessary for neuroinflammation-induced Ido induction (10)], full induction *in vivo* requires synergy with glucocorticoids or Gal-9. As Gal-9 is expressed in many cell types within the brain, expression and induction of hippocampal Gal-9 can occur *via* activation of cells resident to the brain. Gal-9 can bind to many cell surface partners including the T-cell immunoglobulin and mucin-domain containing-3 (TIM-3) receptor, as well as directly associating with several transcription factors (51) within the cell. Further analysis is required to define which of these possible mechanisms are responsible for Gal-9’s ability to enhance IFNγ-induced DO expression.

There are multiple autoimmune disorders associated with elevated levels of both Ido1 and Gal-9 (32, 34–38). As Ido2 function is associated with the development of severe rheumatoid arthritis symptoms (52), Gal-9’s ability to enhance Ido2 expression may promote the development or severity of symptoms of rheumatoid arthritis or other autoimmune conditions. Thus, further characterization of the synergy between IFNγ and Gal-9 during the induction of Ido1 and Ido2 is important to our understanding the pathophysiology of autoimmune disorders such as MS, Hashimoto’s disease, or rheumatoid arthritis. Similarly, Gal-9 is an important regulator of the immune system, promoting the differentiation of T regulatory cells (53) and modulating viral pathogenesis (54). Ido1 plays a similar role in T cell differentiation (55) and also modulates viral pathogenesis (56). These findings open that possibility that one of the mechanisms by which Gal-9 controls T cell differentiation and viral pathogenesis is its ability to direct DO expression. These links warrant further investigation.

### Tdo2

Three Tdo2 transcripts are expressed in the mouse brain, only Tdo2-FL is enriched in hippocampus (39, 57). Similar to Ido1 and Ido2, this specific enrichment implies a distinct regulatory mechanism for transcript expression. Although Tdo2-v1 and Tdo2-v2 expression was increased in the hippocampus post-pI:C administration to mice, treatment of OHSCs with pI:C, pro-inflammatory cytokines or LPS (39) did not increase Tdo2 expression. Thus, another factor(s) must mediate increased Tdo2 expression *in vivo*. Using OHSCs, Tdo2-v1 and Tdo2-v2 expression was increased by Gal-9. Since pI:C was a more potent inducer of hippocampal Gal-9 compared to LPS *in vivo*, the ability of pI:C administration to increase Tdo2-v1 and Tdo2-v2 expression may be mediated by central Gal-9 induction. In contrast to Tdo2-v1 and Tdo2-v2, Tdo2-FL expression was increased by glucocorticoids (Dex and corticosterone) in OHSCs [Figure 8; (39)], but not cytokines or Gal-9. Thus, like the Ido’s, Tdo2 evolved to permit differential transcript expression in a stress- and inflammation-specific manner within the brain. Tdo2-FL expression is dependent on glucocorticoid activity; whereas Tdo2-v1 and Tdo2-v2 are responsive to Gal-9. These data suggest that different promotors used for Tdo2 induction (58) are necessary to meet the increased metabolic (NAD^+^) demand (Figure 10) associated with stress (Tdo2-FL) and neuroinflammation (Tdo2-v1 and Tdo2-v2), as previously suggested (44–46). A repercussion of aberrant Tdo2 expression may be altered behavior such as those associated with schizophrenia (59, 60) and anxiety (61, 62).

## Conclusion

This is the first manuscript to link Gal-9 to the *Kynurenine Pathway*. Following LPS and pI:C administration, IFNγ and Gal-9 expression are induced within the mouse hippocampus, as are Ido1 and Ido2. Using OHSCs, we clearly show that these two inflammatory modulators act in synergy to increase Ido1 and Ido2 expression. This *ex vivo* synergism presumably has a role during *in vivo* induction of Ido1 and Ido2 (Figure 10). Since changes in Gal-9, IFNγ, and Ido1 expression have been independently linked to central diseases such as depression and MS (2, 32, 34, 63), the ability of Gal-9 to accentuate DO expression during neuroinflammation may play a significant role in these and other psychiatric conditions and should be further studied as a putative therapeutic target. Finally, the action of Gal-9 is highly specific to the induction of distinct Ido1, Ido2, and Tdo2 transcripts, suggesting complex posttranslational control over DO expression. Our understanding of this process is in its infancy. Additional work is required to clarify the relevance of DO expression to psychiatric illness, autoimmune disorders, and neurological disease.

## Author Contributions

AB, ML, AS, and RM contributed to the conception of work plus acquisition, analysis, and interpretation of data. JR, KY, and TJ contributed to acquisition, analysis, or interpretation of data. All authors contributed to drafting the manuscript and approve the final version. All authors accept accountability for the content of the work.

## Conflict of Interest Statement

The authors declare that the research was conducted in the absence of any commercial or financial relationships that could be construed as a potential conflict of interest.
